# The Journey of Addiction: Barriers to and Facilitators of Drug Use Cessation among Street Children and Youths in Western Kenya

**DOI:** 10.1371/journal.pone.0053435

**Published:** 2013-01-09

**Authors:** Lonnie Embleton, Lukoye Atwoli, David Ayuku, Paula Braitstein

**Affiliations:** 1 Department of Medicine, College of Health Sciences, School of Medicine, Moi University, Eldoret, Kenya; 2 Department of Mental Health, College of Health Sciences, School of Medicine, Moi University, Eldoret, Kenya; 3 Department of Behavioral Sciences, College of Health Sciences, School of Medicine, Moi University, Eldoret, Kenya; 4 Department of Medicine, School of Medicine, Indiana University, Indianapolis, Indiana, United States of America; 5 Dalla Lana School of Public Health, University of Toronto, Toronto, Ontario, Canada; 6 Regenstrief Institute, Inc., Indianapolis, Indiana, United States of America; Vanderbilt University, United States of America

## Abstract

This mixed-methods study examined barriers to and facilitators of street children’s drug use cessation in Eldoret, Kenya utilizing a cross-sectional survey and focus group discussions with a community-based sample of street-involved children and youth. The primary objective of this study was to describe factors that may assist or impede cessation of drug use that can be utilized in developing substance use interventions for this marginalized population. In 2011, 146 children and youth ages 10–19 years, classified as either *children on the street* or *children of the street* were recruited to participate in the cross-sectional survey. Of the 146 children that participated in the survey 40 were invited to participate in focus group discussion; 30 returned voluntarily to participate in the discussions. Several themes were derived from children’s narratives that described the barriers to and facilitators of drug cessation. Specifically, our findings reveal the strength of the addiction to inhalants, the dual role that peers and family play in substance use, and how the social, cultural, and economic context influence or impede cessation. Our findings demonstrate the need to integrate community, family and peers into any intervention in addition to traditional medical and psychological models for treatment of substance use dependence.

## Introduction

Tens of millions of children around the world find themselves living or working in the streets [1], and in Kenya, it is estimated that 250,000 to 300,000 children are street-involved [2]. Street children are faced with a myriad of challenges in their daily lives, including child abuse and exploitation [3], [4]. Particularly in resource-constrained settings [5] there are very few support systems or programs to assist them. Often, these children lack a balanced social network, and do not have an adequate relationship with an adult caregiver, leaving them extremely vulnerable with many of their physical, mental, and social needs unfulfilled [6]. In turn, these marginalized children fall into patterns of drug use in order to cope with their adverse circumstances and survive on the streets [7].

The prevalence of substance use by street-involved children and youth in low-to-middle income countries has been well documented. Lifetime use of substances ranges between 14% and 92% as reported in studies from Latin America, Africa, the Middle East and Asia [8], [9], [10], [11], [12], [13], [14]. Street children’s drug use often commences with alcohol, tobacco and inhalants which are legal and easily accessible in most countries [3], [4], [12], [15], [16], [17], [18], [19], [20], [21]. The use of inhalants in the form of sniffing glue is particularly prevalent among street children around the world in a variety of cultures and contexts. The pervasive use of glue and other inhalants has been reported in studies from Latin America, Africa, the Middle East and Asia [3], [4], [16], [17], [20], [21], [22], [23], [24], [25], [26]. A variety of reasons are cited for using inhalants and other substances, but there are similarities amongst street children and youth in resource-constrained settings: children often report that using substances assists them in forgetting their problems, dulling hunger, gaining peer acceptance, feeling warmer, and enduring difficult work [3], [14], [16], [17], [18], [21], [24].

Few studies have documented street children’s attitudes or perceptions towards drug use, their awareness of their own misuse and desire to quit. Publications suggest that street children have a moderate degree of awareness about the negative health outcomes associated with their substance use, yet they continue to engage in use [10], [14], [18], [21], [27], [28]. Gaining a greater understanding about their perceptions of their inhalant and other drug use, and the factors that enable or impede street children’s substance use and its cessation in resource-constrained settings is paramount to designing and implementing harm reduction and rehabilitation interventions for this vulnerable population. This mixed-methods study examined barriers to and facilitators of street children’s inhalant and other substance use cessation in Eldoret, Kenya utilizing a cross-sectional survey and focus group discussions (FGD) with a community-based sample of street-involved children and youth ages 10–19 years. The primary objective of this study was to describe factors that may assist or impede cessation of inhalant and other substance use that can be utilized in developing substance use interventions.

## Methods

### Study Design

We employed a mixed-methods study design to elicit street children and youth’s perceptions of substance use cessation and identify factors that may assist or impede quitting. We utilized FGD for qualitative descriptive inquiry and a cross-sectional survey that was designed within the Knowledge, Attitudes and Practices (KAP) model. This approach allowed us to clearly understand street children’s perceptions and attitudes towards substance use cessation within the context of their experiences on the streets.

### Study Setting

Eldoret is a town in western Kenya and is the administrative centre of the Uasin Gishu County. It is home to Moi University (including Kenya’s second medical school), Moi Teaching and Referral Hospital, and the USAID-AMPATH Partnership [24], [29]. With a total population of 289,380, it is currently the 5th largest city in the country [30] and is located within the most rapidly urbanizing region of the world [31]. Rapid urbanization and rural-to-urban migration has resulted in the development of many informal settlements surrounding the town. 51.3% of the population in Uasin Gishu County live below the poverty line and approximately 52% of the population are below the age of 20 [30].

Post-election violence, rapid urbanization, abject poverty, and HIV/AIDS have contributed to the existence of children on the streets of Eldoret [24], [28], [32], [33]. Street children in Eldoret were first reported in 1989, with numbers increasing substantially around 1991–1992 and 2007–2008 in the wake of post-election violence due to internal displacement that resulted in large numbers of families migrating into impoverished urban slums surrounding the town [6], [24], [28]. Displacement for many families resulted in loss of property, thereby leaving them destitute [6]. Many children living in poverty stricken settlements became street-involved, due to the difficult living situations they found themselves in [6], [32].

### Study Population

#### Eligibility

Street-involved children and youth were eligible to participate if they were between the ages of 10 to 19 and not currently enrolled in an educational institution and either a) spending a portion or majority of their time on the street working or roaming while returning to sleep with family or a guardian at night (*child on the street*) or b) having limited or no contact with family and spending both days and nights living and sleeping on the streets or in a communally rented shelter (*child of the street*).

#### Recruitment and enrolment

Extensive street outreach and study sensitization occurred in the “bases/barracks” (primary locations in which street children reside) in Eldoret town. An existing relationship between the research team and a number of street-involved children and youth in Eldoret assisted in identification and outreach within these locations. A street outreach worker with extensive experience working with this population in Eldoret was engaged to conduct the outreach and provide information about the study. Prior to recruitment, the street outreach worker with one of the investigators (LE) visited all of the sites throughout the town known to be street children’s bases, as well as to organizations providing services to children in street circumstances to identify all possible locations where street children are found. Both convenience and snowball sampling methodologies were utilized to recruit participants. During outreach sessions, conducted both individually and with groups in locations where street children reside, the purpose of the study was explained and children were invited to participate voluntarily in the investigation. The street outreach worker would regularly re-visit bases throughout the study to recruit children interested in participating. An attempt was made to recruit equal numbers of children from different bases to obtain a representative sample. Eligible children and youth were also identified and recruited through their peers using snowball sampling. Children that had completed the survey were asked if they had any friends on the street that met our eligibility criteria who would be willing to participate. If they did, they were invited to bring them to the study clinic to participate voluntarily. The survey was conducted at a study clinic on the grounds of MTRH that is dedicated to research with vulnerable children.

Children and youth were purposively recruited for FGD when participating in the survey based on their age and sex and the target group required to reach the desired sample. Participants were given a date and time to return to attend a one to one and a half hour discussion with their peers. The study clinic provided a neutral, safe, and private location that street-involved children felt comfortable attending to discuss substance use. Children and youth gave their assent and enrolled via the project social worker at a study clinic for both the survey and FGD.

### Sample Size

We determined the need to create four distinct groups for FGD with a mix of both *on the street* and *of the street* children stratified by age and sex. These consisted of the following four distinct groups: 1) 15–19 years of age and male, 2) 15–19 years of age and female, 3) 10–14 years of age and male, 4) 10–14 years of age and female. We determined the need to perform two focus group discussions in each distinct category with 8 participants in each in order to compare and contrast data between the divisions as well as to ensure adequate data is collected on a diversity of attitudes and beliefs. There were therefore a total of 64 participants required.

### Protection of Human Subjects

This study received ethical approval from the Indiana University Institutional Review Board, the University of Toronto Research Ethics Board, and the Moi University/Moi Teaching and Referral Hospital Institutional Research Ethics Committee. Approval for the study was also provided by the District Children’s Officer, and we obtained a waiver of individual guardian consent because as per human subjects regulations, the study was minimal risk, the study could not have been practicably carried out without the waiver, and because the waiver did not adversely alter the risk-benefit ratio for participants. Individual written assent was obtained from each participant. Assent was obtained by a social worker trained in assenting vulnerable populations (especially children). Children requesting or requiring healthcare but who were not eligible to participate in the study or who refused to provide assent were provided with healthcare services without enrolment into the study.

### Data Collection

#### Survey

The survey was conducted in Kiswahili through face-to-face interviews using a questionnaire comprised of 78 questions. Prior to the study commencing the survey was translated into Kiswahili. The survey and FGD were conducted in Swahili language which is Kenya’s national language due to the various backgrounds and locations street children come from. However, many street-involved children and youth utilize a street language known as ‘*Sheng*’ and do not adequately comprehend conventional Kiswahili due to their absence from primary school. Therefore, the questionnaire was administered at the study clinic located at MTRH by a trained (Kenyan) research assistant (fluent in English, Kiswahili and *Sheng*) and the principal investigator using oral translation from English into Kiswahili/*Sheng*. Responses and interview data were recorded in English on the questionnaire by the research assistant. A preliminary version of this questionnaire was piloted using 52 questions at a street children’s drop-in centre in Eldoret, Kenya to evaluate changes in knowledge, attitudes and practices of street-involved children and youth participating in a substance abuse curriculum. The questionnaire was subsequently modified to ascertain additional in-depth information concerning substance use practices and demographics of the participants.

#### Focus group discussions

FGD were conducted in Kiswahili/*Sheng* in a private space at the study clinic located at MTRH and facilitated by the research assistant with the assistance of a street youth outreach worker as a co-facilitator in the presence of the principal investigator. The facilitator used a structured interview guide consisting of 12 open-ended questions to lead the discussion that lasted 1 to 1.5 hours. With the participants’ permission each of the focus group discussions were captured with a digital audio recorder for transcription and translation purposes and notes were recorded by the co-facilitator.

### Data Analysis

#### Qualitative

**Figure 1:**
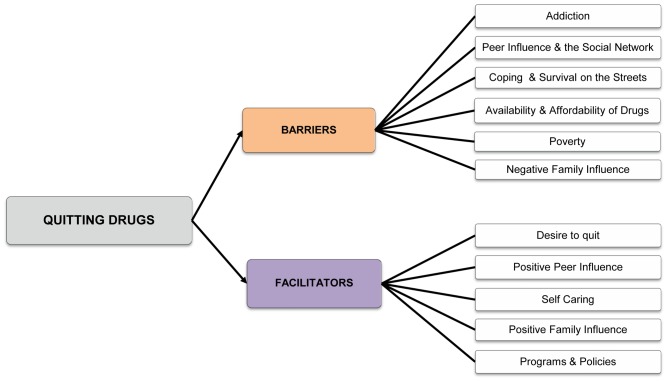
Themes derived from thematic content analysis.

Data were captured using a digital audio recorder and upon completion of all focus group discussions was transcribed and translated into English. Thematic content analysis was conducted and data were coded according to themes and patterns that emerged until saturation was reached using NViVo (version 9.0, QSR International, Australia). Themes in association with barriers and facilitators were derived from street children’s narrative in response to the structured interview guide. The themes derived are presented in Figure 1.

#### Quantitative

Data were entered into Epi Info (3.5.1) from paper-based surveys and subsequently imported into SAS (version 9.0, Institute Inc, Cary, NC) for analysis. Frequencies and percents were calculated for selected variables in relation to qualitative themes that emerged in order to describe substance use and cessation.

## Results

There were 151 street-involved children and youth recruited; 5 were ineligible to participate in the study as they didn’t meet the inclusion criteria. There were therefore 146 children and youth aged 10–19 classified as either *children on the street* or *children of the street* enrolled to participate. Detailed socio-demographics of the study population have been published elsewhere [14]. Briefly however, there were, 98 (67%) participants classified as *children of the street* and 48 (33%) as *children on the street*. Males accounted for the majority of participants in both categories (of the street: 85% and on the street: 65%). The median age of participants was 14 years (IQR 12–16). Of the 146 children that participated in the cross-sectional survey 40 were invited to participate in FGD; 30 returned voluntarily to participate in a FGD according to age and gender. There were therefore two FGD conducted with 12 boys aged 10–14, two with 13 boys 15–19 and one with five girls aged 15–19. The participants that did not return to participate in FGD were primarily girls between the ages of 10–14 and 15–19.

As previously reported [14], substance use is a major problem amongst street children and youth in Eldoret, Kenya. Specifically, 74% (n = 108) of participants had any lifetime drug use, with 83% (n = 81) of *children of the street* having ever used in comparison to only 56% (n = 27) of *children on the street* [14]. Of those with a history of lifetime drug use, 83% (n = 90) were currently using. The most commonly used substance amongst lifetime users was glue (67%, n = 98) followed by alcohol (47%, n = 69), cigarettes (45%, n = 65), miraa (a plant that contains the active ingredient cathinone and acts as a stimulant when chewed) (33%, n = 48), marijuana (29%, n = 42), petrol (24%, n = 35) and pharmaceuticals (8%, n = 11) (Table 1). Of those currently using drugs, 94% reported sniffing glue within the last 30 days and 78% admitted to using daily, demonstrating the overwhelming use and dependence on glue.

**Table 1:** Prevalence of lifetime and current drug use amongst a 146 street-involved children and youth in Eldoret, Kenya.

	TOTAL n (%) N = 146	CHILD ‘OF’ n (%) Total (n = 98)	CHILD ‘ON’ n (%) Total (n = 48)
**Lifetime Drug Use**			
Yes	108 (74.0)	81 (82.7)	27 (56.3)
No	38 (26.0)	17 (17.4)	21 (43.8)
**Drugs Ever Used**			
Glue	98 (67.1)	74 (75.5)	24 (50.0)
Alcohol	69 (47.3)	52 (53.1)	17 (35.4)
Cigarettes	65 (44.5)	51 (52.0)	14 (29.2)
Miraa	48 (32.9)	37 (37.8)	11 (22.9)
Marijuana	42 (28.8)	29 (29.6)	13 (27.1)
Petrol	35 (24.0)	27 (27.6)	8 (16.7)
Other Pharmaceuticals	11 (7.5)	9 (9.2)	2 (4.2)
**Currently Using Drugs**			
Yes	90 (61.6)	69 (70.4)	21 (43.8)
No	56 (38.4)	29 (29.6)	27 (56.2)

## Barriers to Cessation

### Addiction

“A new person can stay for one month in town without sniffing the glue after which they crave, taste and begin using it. The journey of addiction starts off…”

(Girl, 15–19)

Street children and youth in Eldoret are aware of the addictive properties of sniffing glue and other substances. The majority of survey participants (85%, n = 124) responded that glue was addictive and 68% (n = 99) thought that all drugs were addictive (Table 2). When non-users and users were asked what they perceived to be the primary reasons street children in Eldoret use substances, addiction was cited for glue (2%, n = 3), tobacco (19%, n = 28), alcohol (4%, n = 6) and marijuana (3%, n = 4). In FGD, the powerful “thirst” to sniff glue was described by many participants. As one participant accounted:

**Table 2:** Barriers to drug cessation among 146 street-involved children and youth in Eldoret, Kenya.

THEME	BARRIER	TOTAL n (%) N = 146	CHILD ‘OF’n (%) Total(n = 98)	CHILD ‘ON’ n (%) Total (n = 48)
ADDICTION	**Glue is addictive**			
	Yes	124 (84.9)	83 (84.7)	41 (85.4)
	No/Don’t Know	22 (15.1)	15 (15.3)	7 (14.6)
	**All drugs are addictive**			
	Yes	99 (67.8)	62 (63.3)	37 (77.1)
	No/Don’t Know	47 (32.2)	36 (36.7)	11 (22.9)
PEER INFLUENCE	**I use glue because my friends do** a,b			
	Strongly Agree/Agree	79 (84.0)	60 (84.5)	19 (82.6)
	Strongly Disagree/Disagree	15 (16.0)	11(15.5)	4(17.4)
	**Who introduced to drug use** c			
	Friend	77 (71.3)	54 (66.7)	23 (85.2)
	Barracks/Base leader	2 (1.9)	1 (1.2)	1 (3.7)
FAMILY INFLUENCE	Family Member	19 (17.6)	17 (21)	2 (7.4)
	Other	10 (9.3)	9 (11.1)	1 (3.7)
	**Family member alcohol, tobacco & other drug use**			
	Yes	115 (78.8)	76 (77.6)	39 (81.3)
	No/Don’t Know	31 (21.2)	22 (22.5)	9 (18.8)
COPING& SURVIVAL	**Glue Helps me cope with reality** b			
	Strongly Agree/Agree	70 (71.4)	52 (70.3)	18 (75.0)
	Strongly Disagree/Disagree	28 (28.6)	22 (29.7)	6 (25.0)

a 4 missing responses.

b Of those who had ever sniffed glue (n = 98).

c Of those who had ever used (n = 108).

“It’s difficult to stop. They are so addicted because they always have the glue by the mouth [respondent demonstrates how the glue is constantly near the mouth and it’s covered by the cuffs of the different garments that the children have on]. If you stop sniffing in the evening for a short time immediately you feel thirsty and start sniffing.” (Boy, 10–14)

In response to inquiries regarding the experiences of those participants that have attempted to stop, many described difficulties due the strong desire to sniff. Many children discussed an intense craving for the glue when attempting to stop, returning home, or being placed in an institution, as one boy stated: “I care and I have tried to stop using it but in vain. I have tried to stop using it but it has been so hard. Recently I was taken to the Rescue Centre (temporary shelter) but when I remembered about the glue, I came back to town” (Boy, 15–19). The desire to sniff was described as so strong that they would forgo buying food when hungry: “If you are hungry and feel the thirst for the glue….when given money most of the times you rush to buy glue instead of food” (Boy, 10–14).

Both boys and girls described withdrawal symptoms experienced when stopping and the on-going cycle of use. Various maladies associated with stopping were described including, feeling restless, unable to sleep, shivering, and headaches that subsided when commencing sniffing again. Two participants offered:

“You know when you stop taking drugs after you were addicted to them you may experience some illnesses like shivering…” (Boy, 10–14)

“When someone is used to taking that glue all the time, stopping is hard…. If that stuff enters your blood… and you stop using it, you get headaches and become sick. It’s an ongoing cycle.” (Girl, 15–19)

#### Peer influence & social network

Peer influence and belonging play a major role in initiation into and on-going use of drugs for street children. The majority of glue using survey participants responded that they strongly agreed or agreed that they use glue because their friends do (84%, n = 79) and were first introduced to using drugs by a friend (71%, n = 77) (Table 2). The process of initiation into street culture involves the introduction to sniffing glue by peers, followed by acceptance and belonging into a social network on the streets.

“When you are a visitor you will not start using glue immediately. At first you will shun the glue and then start using it slowly by slowly until you get used to it. You will get introduced to it by friends through sharing what they already have.” (Boy 10–14)

Some children described feeling being tricked or cheated into using glue by their peers who told them it would help them sleep and reduce stress. As one boy explained: “They cheat you into use of the drugs” (Boy 10–14). Additionally children explained how older street youth, drug dealers and gang leaders force children to buy drugs even when they no longer want to. One girl explained the brutality suffered by those who do not submit to using:

“He forces you to sniff that glue and if you don’t he beats you up or looks for other boys to molest you… [*Respondents in agreement*]…. When [drug dealer’s name] wants you to sniff glue, he gives you glue worth twenty shillings. Eventually you will have to pay a thousand shillings in kind but not in monetary form.” (Girl 15–19)

Furthermore, one girl described the inability to quit using as long as her friends are using it, “They cannot stop because their friends still sniff it. If the friends stop sniffing, they will feel it and opt to stop sniffing” (Girl, 15–19).

### Coping & Survival on the Streets

“At night they sniff glue and it gives them warmth. They view the glue as a ‘blanket’ to prevent them from feeling cold. That glue provides warmth.” (Boy 10–14)

Glue and other substances provide street children and youth with coping mechanisms for the harsh reality of life on the streets. 71% (n = 70) of children surveyed who had ever used glue (n = 98) strongly agreed or agreed that glue helps them cope with reality (Table 2). When non-users and users were asked what they perceived to be the primary reasons street children in Eldoret use substances, to feel warmer was cited by 27% (n = 40) of subjects in relation to glue and to gain strength was cited by 29% (n = 42) in relation to marijuana. Focus group participants explained that using substances reduced hunger, assisted with sleeping, reduced stress, helped them to forget the past, and gave strength and courage to fight, steal food and survive.

“People sniff glue because of the problems they go through. Sniffing of the glue depends on the situation one is going through. You may find one has slept out in the cold and they take glue because it makes them high, warm and as a result they don’t feel cold. Others smoke bhang [marijuana] so that they can get strength to work. It makes them do a lot of work. Everything has its advantages.” (Boy 15–19)

#### Availability & affordability

The widespread availability, affordability, and legal status of glue make it the drug of choice for many street children. As one boy explained: “It’s most common, plenty and readily available in town” (Boy, 15–19). Children discussed the past transition between petrol to glue due to the increasing price of petrol in comparison to glue. Many participants discussed that as long as cobblers and drug dealers have access to glue and it remains legal, street children will not be able to stop using, “There is no way I can stop taking drugs yet the drug seller still sells them” (Boy 15–19). One boy described a solution to the availability of glue:

“They should stop the drug sellers…. Drug sellers should be arrested…. Especially the cobblers, because they know it is wrong yet they sell them to the street kids…. You know they may prevent the shop drug sellers [glue sellers] from selling it to the street kids, but they would still sell to the cobblers who in turn sell to the street kids” (Boy 10–14)

#### Negative family influence

Street children surveyed responded that 79% (n = 115) had a family member who used alcohol, tobacco or other drugs and 18% (n = 19) of children who ever used drugs (n = 108) were introduced by a family member (Table 2). Children in the FGD described parents who had purchased glue for them, and brewed and consumed alcohol creating a negative family environment. One boy explained the influence of a family member brewing alcohol on a child’s initiation to substance use:

“For those who leave their homes, they learn it from their parents. For example when the mother brews alcohol, why can’t the child drink? The child has to drink because he sees his parents brew. Such a child would think, “Let me taste it because I see my father and mother use it”…. When he tastes it on the first and second day…you know starts that way, you may taste a half a glass then think, “Let me add a glass tomorrow” then one gets used to it.” (Boy 15–19)

#### Poverty

Uasin Gishu County and Eldoret town are subject to high levels of poverty with approximately half the population living below the Kenyan poverty line [30]. Many children living in informal settlements surrounding the town become street-involved, due to the difficult living situations they find themselves in. Additionally, children living in rural settings outside of the county often migrate to Eldoret in search of improved living circumstances. Children and youth turn to the streets to support themselves or make an economic contribution to the family, as explained by one girl: “Some children have left their homes because of poverty. Because of some parents giving up with life, they tell the child to go away and fend for themselves” (Girl 15–19). In the process of becoming street-involved, children and youth are introduced to street culture and substance use, “We have left our homes because of problems and have resorted to sniffing glue” (Girl 15–19). One girl recounted how her impoverished situation at home led her to the streets and drug use:

“My mother has a problem. My father was a polygamist and when he died my mother had to be on her own. She resulted into taking alcohol, wasn’t feeding us or taking care of our needs. We were suffering a lot. I heard people talk of a place called Town and street children. One day I met the street children and I followed them to the market where we ate so many bananas until I was satisfied. In the process they were sniffing glue so I made up my mind to stay in town” (Girl 15–19)

### Facilitators of cessation

#### The Desire to quit

Many participants expressed the desire to quit using glue and other drugs. 94% (n = 85) of current users responded that they wanted to quit using drugs, while 85% (n = 90) of ever users had tried to quit in the past, yet only 57% (n = 60) had sought help for their drug use (Table 3). As expressed by one boy: “I really want to stop using it and in future I will stop” (Boy 10–14). Although many participants have the desire to quit, many surveyed (47%, n = 68) felt that it was too hard to stop using drugs.

**Table 3:** Facilitators of drug cessation among 146 street-involved children and youth in Eldoret, Kenya.

THEME	FACILITATOR	TOTAL n (%) N = 146	CHILD ‘OF’ n (%) Total (n = 98)	CHILD ‘ON’ n (%) Total (n = 48)
DESIRE TO QUIT	**Do you want to stop using drugs?** a			
	Yes	84 (94.4)	64 (94.1)	20 (95.2)
	No	5 (5.6)	4 (5.9)	1 (4.8)
	**Have you tried to quit drugs?** b			
	Yes	90 (84.9)	68 (85.0)	22 (84.6)
	No	16 (15.1)	12 (15.0)	4 (15.4)
	**Have you ever sought help for drug use?**			
	Yes	60 (57.1)	43 (54.4)	17 (65.4)
	No	45 (42.9)	36 (45.6)	9 (34.6)
	**It’s too hard to stop using drugs** b			
	Strongly Agree/Agree	68 (47.2)	47 (49.0)	21 (43.8)
	Strongly Disagree/Disagree	76 (52.8)	49 (51.0)	27 (56.2)
	**I will never stop using glue** b			
	Strongly Agree/Agree	18 (18.8)	16 (21.9)	2 (8.7)
	Strongly Disagree/Disagree	78 (81.2)	57 (78.1)	21 (91.3)
SELF CARING	**Do you think you have enough information about drugs?**			
	Yes	54 (37.0)	39 (39.8)	15 (31.3)
	No	92 (63.0)	59 (60.2)	33 (68.8)
	**Ever taught dangers of using drugs**			
	Yes	42 (28.8)	29 (29.6)	13 (27.1)
	No	104 (71.2)	69 (70.4)	35 (72.9)
	**I care about myself and my health**			
	Strongly Agree/Agree	139 (95.2)	93 (94.9)	46 (95.8)
	Strongly Disagree/Disagree	7 (4.8)	5 (5.1)	2 (4.2)
	**I care about my friends and their health**			
	Strongly Agree/Agree	113 (77.4)	77 (78.6)	36 (75.0)
	Strongly Disagree/Disagree	33 (22.6)	21 (21.4)	12 (25.0)
PROGRAMS & POLICIES	**I would like to see more services to help street children stop using drugs**			
	Yes	133 (91.1)	93 (94.9)	40 (83.3)
	No/Don’t know	13 (8.9)	5 (5.1)	8 (16.7)
	**I think the community should be doing more to help street children stop using drugs**			
	Yes	135 (92.5)	92 (93.9)	43 (89.6)
	No	11 (7.5)	6 (6.1)	5 (10.4)

a Of current users (n = 90).

b Missing 2 responses.

#### Positive peer influence

Participants responded favourably to questions regarding peers that do not use drugs and their ability to assist other street children and youth to quit. Positive peer influence was discussed by many participants as a factor that may facilitate quitting drugs. Although some peers play a negative role in on-going substance use, others have the ability to act as role models, provide advice and support to their friends when trying to quit drugs. One boy discussed how his friend who doesn’t sniff glue assists him:

“When we work together and get money, my friend who doesn’t sniff glue decides that we buy food. When we get money my friend decides that we should buy food. He has been a good friend and has given me advice and I appreciate it. I am constantly with him and his help has made me see the sense of stopping to sniff glue slowly by slowly and eventually I will stop completely. This could be another way of one to stop using glue” (Boy, 15–19)

Another girl expressed that their peers that do not use care about them and their health and therefore provide support and advice:

“M advises me to stop sniffing glue…. She says that it is bad…. She cares about my health…. She is concerned about my health because if I die as a result of sniffing glue she will be all alone*…* She will have no friend. She will remain all alone” (Girl, 15–19)

#### Self caring

Participants in both the survey and FGD acknowledged that using substances was bad for their health and could even kill you. Some participants expressed knowledge about the damage that various drugs do to different parts of the body, and that this made them stop. One boy explained that this made him quit smoking:

“No one told me to stop smoking. I read in a book that cigarette destroys lungs. You can get lung cancer from smoking cigarettes. When I thought about it, I decided to stop.” (Boy, 15–19)

Although information about the detrimental health effects of using substances may lead to cessation, very few children surveyed (37%, n = 54) felt that they had enough information about drugs and only 29% (n = 42) of study participants had ever been taught the dangers of using drugs. Importantly, 95% (n = 139) strongly agreed or agreed that they cared about themselves and their health, while 77% (n = 113) strongly agreed or agreed that they cared about their friends and their health (Table 3). Girls in particular were concerned with the effects of substance use on their reproductive health. One girl offered: “I would want to give birth to my own children…. If I sniff a lot of glue I will not bear children” (Girl 15–19).

#### Positive family influence

Many children on the street remain in contact with their families and often return home at night to sleep. When inquiring of FGD participants “How can such parents assist their kids to stop using drugs”, participants discussed the varying ability of parents and families to take care of them and the challenges in the home that led such children to the streets and substance use in the first place.

However, in situations where a child’s parents find themselves in a stable situation one boy offered:

“The easiest way of helping such a kid is getting hold of them and taking them home…. You know, the kid understands his parent and listens to the parent. When you beat up a child you won't help him, it will just be like hurting the kid because you may beat him up and find him using drugs the next day. But when you sit down such a kid and advise him and tell him, “Whatever you are doing is not good and you are spoiling our family name, when neighbours and friends see you going to the streets and taking drugs it creates a bad picture, you just concentrate on education first.” (Boy 15–19)

Another boy recounted how his parents stopped him from using substances despite remaining street-involved:

“My parents stopped me from using the drugs. They told me that they had never seen anyone in our family use glue apart from me. I made a decision to stop using glue, and be obedient to my parents. I stopped using it.” (Boy 15–19)

#### Programs & policies

The majority of street children surveyed (91%, n = 133) indicated they wanted to see more services available to help street children stop using drugs and 93% (n = 135) felt that the community should be doing more to assist (Table 3). However, many in the FGD were in disagreement about the best approach to creating programs and policies aimed at street children and substance use cessation. Participants came up with a variety of suggestions that would assist street children in quitting drugs. Many suggested returning to school, being repatriated home, being taught a skilled trade, and setting up rehabilitation centres. One girl explained the potential benefits of providing training and education:

“Enrol them for some form of training and when they get a good education and job it will make their life better. Such a child will leave the streets and help rehabilitate other street children. He or she can take the street child to school and in future that child will also help others on the street.” (Girl 15–19)

While another girl discussed how children and families could be assisted to facilitate an up-stream intervention to enable families to provide and prevent children from migrating to the streets:

“The street children should be taken back to their homes. For the children whose parents are not able to care for them, they should be assisted. Some businesses should be set up for such parents. They can be given loans and when they stabilize they pay back.” (Girl 15–19)

Children and youth were divided as to whether village elders, Chiefs, and other officials could be of assistance. In response to the question “Should village elders and chiefs assist street children?” girls strongly responded “No *[Respondents answer unanimously]….* In fact it’s the village elders who beat us up thoroughly” (Girl, 15–19). Although, others felt they could assist in creating policy aimed at limiting access to glue in Eldoret:

“Most street children use glue. Imposing a ban on glue would be the best thing to do. A ban should be imposed on the people who sell it. The people who buy that glue and sell it to the street children should be stopped. They should not ban the cobblers but those who sell it to the street children” (Boy 15–19)

However, there was disagreement amongst the group and another participant brought the following to attention:

“There is a time they banned the sale of glue but what people would do is to take money to the cobblers who would buy the glue for “repairing shoes” when in real sense they sell it and it ends up being sold to the street children.” (Boy 15–19)

## Discussion

Our study reveals several important factors that street children recognize as barriers or facilitators to inhalant and substance use cessation that are key to developing programs and interventions aimed at drug use cessation in this marginalized population. Specifically, the dual role that peers and family play in drug use initiation and cessation highlight the importance of integrating peers and family into any intervention strategy due to the strong influence they have in street children’s lives. The addictive properties of glue sniffing and associated withdrawal symptoms demonstrate the need to address physical and mental health outcomes in rehabilitation programs.

Often the first step in drug and alcohol use cessation is acknowledging that a problem exists and expressing a desire to quit [34], [35], [36]. Almost all of the children in this study responded that they wanted to quit using drugs and discussed this desire in the FGD. This is a positive response as research in other inhalant using populations has demonstrated that having motivation to enter treatment decreased the likelihood of relapse [37] and that often volatile solvent users do not present to treatment willingly [38]. This strong desire among street children to quit inhalant use is promising for the design and engagement of children in drug cessation programs, especially in light of the fact that most users do so for coping and survival.

The powerful addiction to glue, withdrawal symptoms, and its ability to pull children back to the streets has not been well documented previously, yet reports of psychological and physical dependence amongst volatile solvent users exist [17], [39]. Additionally, there is evidence of withdrawal symptoms associated with volatile solvent cessation that may last two to five days and include headache, irritability, sleep disturbance, sweating, nausea, and other more severe symptoms [39], [40]. The craving and withdrawal symptoms children have described in our FGD indicate that any substance use cessation intervention needs to address the addictive properties of glue and the physical and mental symptoms experienced while quitting. Without supporting and assisting children succumbing to withdrawal symptoms they are likely to relapse to sniffing in order to negate the poor feelings associated with cessation. Evidence from interventions in other solvent using populations points to the importance of detoxification and support in this process [38], [39].

The role of the peer and social network in street children’s substance use is pivotal and multifaceted. This study reveals that street peers can play both a positive and negative role in impeding or facilitating substance use cessation. Studies in other resource-constrained settings have found that street children obtain support from their street-involved peers, who form a strong social network and sense of belonging [28]. However, these social networks often facilitate initiation and on-going substance use through peer pressure [4], [10], [17], [18], [28], [41]. Our study confirms that peers are involved in substance use initiation and facilitate on-going use of drugs. Additionally, children and youth have revealed that they feel coerced into using by their peers and that older youth, especially older boys, may use force or threaten girls to engage in substance use. Partaking in glue sniffing within street culture in Nairobi, Kenya has been described as a necessity to acceptance and protection within a group on the streets [28]. Due to the strong influence peers play in facilitating drug use, utilizing the peer network may be an effective intervention. Harnessing the power of positive peer influence on the streets by creating a network of former or non-substance using peer outreach workers that support and mentor children and youth to prevent and reduce drug use, implement harm reduction strategies and disseminate education materials has the potential to be highly effective due to the trust, social bond, and influence inherent in their street peers. This is supported by the fact that our study has demonstrated the positive influence street peers can have on their friends in encouraging and facilitating substance use cessation.

Familial discord, alcohol use, poverty, and difficult home circumstances are recurring themes within street children’s accounts of migrating to the streets and reasons for engaging in substance use. Brewing traditional alcohol in Kenya for income-generation in low-income settings is common and is primarily performed by women. Despite being an illegal activity in Kenya, it is often carried out in informal settings, usually in homes [42], [43]. Studies have demonstrated high rates of alcohol consumption and abuse within the country, including among the youth [42], [44]. Research on traditional alcohol brewing and its impact on the family environment in Kenya is limited [42], [43]. Our results show that familial alcohol use and traditional brewing are contributing to street children’s initiation and on-going use of drugs and alcohol, and migration to the streets to improve their living circumstances. Street children recounted being introduced to alcohol in the home by parents who brew, and 18% of children surveyed indicated they were introduced to drugs by a family member. Additionally, the majority of children indicated someone in their family used tobacco, alcohol or other drugs, and this significantly increases the odds of street children using drugs themselves [14]. The description of alcohol abuse and poverty within the home resulting in neglect and difficult living circumstances in combination with the role traditional brewing plays on alcohol initiation demonstrates the need to involve families and the community in any intervention for street children’s drug use cessation and community re-integration. Difficult home circumstances acting as a push factor to the streets has been reported in other resource-constrained settings [18], [45], and it has been suggested that improving conditions in the home through income-generation projects and community development are vital in mitigating street involvement [5]. However, in Kenya it is evident that any intervention also needs to address traditional brewing and familial substance use that strongly influences street children’s engagement in substance use.

We recognize that this study has strengths and limitations. Strengths include that this is one of the only studies to document in detail street children’s perceptions and attitudes towards their drug use and substance use cessation. Secondly, we utilized both qualitative and quantitative research methods that complemented each other to give a comprehensive in-depth understanding of substance use amongst this population. Lastly, our study was able to recruit both boys and girls and children *on* and *of* the street to give a diversity of perspectives.

This study may also have limitations. The non-random recruitment and sampling of participants through active outreach and a snow-ball sampling technique is prone to selection bias. Children and youth who self-selected to participate in the survey through snow-ball sampling and those who accepted to participate versus declined through active outreach could be systematically different. To limit the effects of selection bias, outreach and study sensitization was performed in all areas of Eldoret where street children and youth are found in an attempt to obtain a representative sample generalizable to the population. Moreover, children who agreed to participate and return voluntarily for FGD may have been systematically different than their peers who did not return. Furthermore, social desirability bias could have affected responses to sensitive questions about drug use cessation, attitudes and practices. Children may have responded to questions about drugs with answers that they believed the interviewer or FGD facilitator wanted to hear. However, we believe that this was minimized due to the strong rapport and relationship the research team had with the participants. Additionally, we were unable to achieve the desired number of participants for FGD, especially girls, and this may have limited the ability to identify gender differences in the perceptions towards substance use cessation.

### Conclusion & Recommendations

There are complex factors at play that influence street children’s substance use initiation, on-going use, and impede cessation. The scope of the problem is broad and requires innovative and multifaceted programs to be effective in preventing, reducing and stopping substance use amongst this vulnerable population. Our findings demonstrate the need to integrate community, family and peers into any intervention in addition to traditional medical and psychological models for treatment of substance use dependence. Additionally, due to the complex nature of the street children crisis in Kenya there is a need for researchers, community members, relevant stakeholders, and policymakers to collaborate not only to mitigate substance use in this population, but identify viable alternatives to street life. Future research should aim to gain the perspectives of the community, families of street children and policymakers on how to address these issues within the socio-economic and cultural context of Kenya.
